# Assessment of Methods for the Intracellular Blockade of GABA_A_ Receptors

**DOI:** 10.1371/journal.pone.0160900

**Published:** 2016-08-08

**Authors:** Laura A. Atherton, Erica S. Burnell, Jack R. Mellor

**Affiliations:** 1 Centre for Synaptic Plasticity, School of Physiology, Pharmacology & Neuroscience, University of Bristol, Bristol, United Kingdom; 2 School of Engineering Mathematics, University of Bristol, Bristol, United Kingdom; Dalhousie University, CANADA

## Abstract

Selective blockade of inhibitory synaptic transmission onto specific neurons is a useful tool for dissecting the excitatory and inhibitory synaptic components of ongoing network activity. To achieve this, intracellular recording with a patch solution capable of blocking GABA_A_ receptors has advantages over other manipulations, such as pharmacological application of GABAergic antagonists or optogenetic inhibition of populations of interneurones, in that the majority of inhibitory transmission is unaffected and hence the remaining network activity preserved. Here, we assess three previously described methods to block inhibition: intracellular application of the molecules picrotoxin, 4,4’-dinitro-stilbene-2,2’-disulphonic acid (DNDS) and 4,4’-diisothiocyanostilbene-2,2’-disulphonic acid (DIDS). DNDS and picrotoxin were both found to be ineffective at blocking evoked, monosynaptic inhibitory postsynaptic currents (IPSCs) onto mouse CA1 pyramidal cells. An intracellular solution containing DIDS and caesium fluoride, but lacking nucleotides ATP and GTP, was effective at decreasing the amplitude of IPSCs. However, this effect was found to be independent of DIDS, and the absence of intracellular nucleotides, and was instead due to the presence of fluoride ions in this intracellular solution, which also blocked spontaneously occurring IPSCs during hippocampal sharp waves. Critically, intracellular fluoride ions also caused a decrease in both spontaneous and evoked excitatory synaptic currents and precluded the inclusion of nucleotides in the intracellular solution. Therefore, of the methods tested, only fluoride ions were effective for intracellular blockade of IPSCs but this approach has additional cellular effects reducing its selectivity and utility.

## Introduction

Network activity supporting cognitive processes within the brain involves a delicate balance between excitation and inhibition [1–7] and the precise control of spike timing in excitatory and inhibitory neurons [5, 8–11]. Perturbations to the excitatory-inhibitory balance within a controlled range can modulate the gain and dynamic range of information processing [12–15], and if uncontrolled can lead to epileptiform activity [16, 17].

To understand how excitation and inhibition interact during network activity, it is often necessary to isolate one from the other. This is challenging because inhibition of either excitation or inhibition disrupts ongoing network activity. Pharmacological approaches using application of GABAergic antagonists to block inhibitory synaptic transmission typically affect the entire preparation being studied which can lead to runaway excitation, the cessation of physiologically relevant network oscillations and the instigation of epileptiform activity [18–21]. One approach to avoid this has been to locally apply antagonists onto the region of interest [22–26] but a precise control over the size of the affected area is difficult to obtain and therefore it is hard to assess the disruption to the wider network activity. Alternatively, specific populations of interneurons may be activated or silenced optogenetically by targeting optically sensitive actuators or inhibitors [7, 27, 28] to subtypes of interneurons via the Cre-recombinase system [29–31]. Whilst this has the potential to achieve a localised and targeted control of inhibition, network activity is still likely to be disrupted within that region. For example, optogenetic silencing of parvalbumin positive interneurones has been shown to reduce the power of gamma oscillations [32] and interrupt or abolish hippocampal sharp wave ripples [24, 33].

For a more localised blockade of inhibition, and particularly for studying the local effects of excitation and inhibition on single cells during ongoing network activity, it is desirable to block inhibition onto an individual cell. To achieve this, one approach is to voltage-clamp a neuron at the inhibitory reversal potential. However inhibitory inputs impinge onto many different locations on the neuronal arbor, and so space clamp issues [34, 35] can preclude the blockade of inhibitory inputs at more distal dendrites. Similarly, holding cells at the inhibitory reversal potential does not prevent the effect of shunting inhibition [36, 37] which can, amongst other things, affect cellular behaviour during ongoing oscillatory activity [38, 39]. To circumnavigate these issues, agents may be included in the intracellular recording solution to diffuse into the cytoplasm and block GABA_A_ receptors. A series of molecules have previously been used, ranging from GABA_A_ receptor pore-blockers such as picrotoxin [25, 40–47] to disulphonic stilbene derivatives such as 4,4’-dinitro-stilbene-2,2’-disulphonic acid (DNDS) [48–53] and 4,4’-diisothiocyanostilbene-2,2’-disulphonic acid (DIDS) [53–56]. However, no comparison of the relative effectiveness of these different agents has been made. To this end, these agents were re-examined for their efficacy in blocking both evoked inhibitory postsynaptic currents (IPSCs) and spontaneously occurring IPSCs during hippocampal sharp waves.

## Materials and Methods

### Ethics statement

All experiments were performed in accordance with the UK Animal Scientific Procedures Act (1986) and local guidance from the Home Office Licensing Team at the University of Bristol. The protocol was approved by the Animal Welfare and Ethics Review Board at the University of Bristol (Home Office Licence Number 30/3207). All surgery was performed under sodium pentobarbital anaesthesia, and all efforts were made to minimize suffering.

### Slice preparation

**Fig 1:**
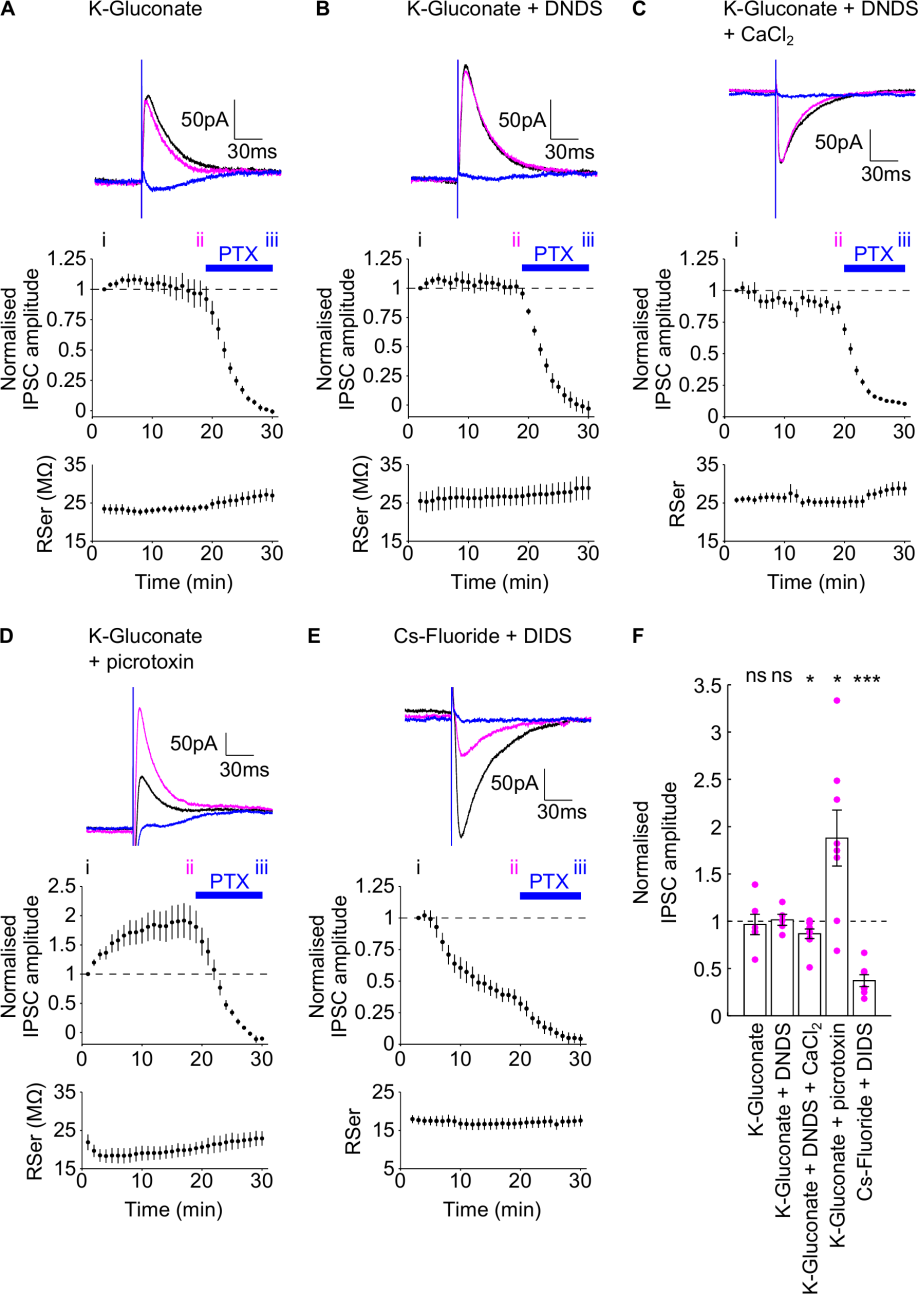
Effect of DNDS, picrotoxin and CsF-DIDS on evoked IPSCs. Upper panels of A-E: Example traces during the diffusion of K-Gluconate (A), K-Gluconate +DNDS (B), K-Gluconate +DNDS & CaCl_2_ (C), K-Gluconate + picrotoxin (D) and Cs-Fluoride +DIDS (E) intracellular pipette solutions. Each trace shows the IPSC at the start of recording (black, time point i in middle panels), in the last minute before bath application of picrotoxin (magenta, time point ii in middle panels) and at the end of the bath application of picrotoxin (blue, time point iii in middle panels). Middle panels of A-E: Normalised IPSC amplitudes. Blue bar indicates the presence of bath picrotoxin (PTX). Lower panels of A-D: Corresponding series resistance during experiments. NBQX and AP5 were present throughout the experiments. E) Group data of the normalised evoked IPSC amplitudes for the four intracellular pipette solutions, taken at time point ii, individual data points marked as magenta dots. Summary statistics represent tests comparing the normalised evoked IPSC amplitudes at time point ii with the normalised baseline amplitude for each data set. Data are plotted as mean ± SEM.

Transverse mouse hippocampal slices were prepared from male C57BL6 mice (4–8 weeks old). Mice were anaesthetised with sodium pentobarbital (300mg/kg i.p.) and transcardially perfused with 20ml of ice-cold cutting solution, containing (in mM): 205 sucrose, 2.5 KCl, 26 NaHCO_3_, 0.5 CaCl_2_, 5 MgSO_4_, 1.25 NaH_2_PO_4_ and 10 glucose, equilibrated with 95% CO_2_ and 5% O_2_. Using this solution, hippocampi were then dissected and mounted onto agar. 500μm thick slices were cut using a VT1200 vibratome (Leica). Following preparation, slices were incubated for at least 2 hours in recording artificial cerebrospinal fluid (aCSF), containing (in mM): 119 NaCl, 10 glucose, 26 NaHCO_3_, 2.5–3.5 KCl, 1 NaH_2_PO_4_, 2.5 CaCl_2_, 1.3 MgSO_4_, equilibrated with 95% CO_2_ and 5% O_2_. During incubation, slices were either stored at 34°C for 30 minutes and then kept at room temperature (Fig 1E), or they were held in an interface chamber at 32°C until use.

### Electrophysiological recordings

For recording, slices were placed into a submerged recording chamber (RC-27L, Harvard apparatus) and maintained at 32°C, with recording aCSF perfused above and below the slice at either 6ml/min (for recordings during spontaneous sharp waves) or 1.5ml/min (for all other experiments). Slices were visualised with infrared differential interference contrast microscopy on a Scientifica SliceScope microscope.

Patch pipettes (4-5MΩ) were pulled using borosilicate glass capillaries (Harvard Apparatus) on a PC-10 vertical puller (Narishige) and were either filled with recording aCSF (for extracellular recordings), or one of the intracellular pipette solutions described in Table 1 (for patch recordings). For DIDS based intracellular pipette solutions, DIDS was dissolved in 0.1M potassium bicarbonate prior to dilution into the pipette solution. The pH of intracellular solutions was adjusted to 7.35–7.4 with KOH (for potassium based intracellular pipette solutions) or CsOH (for the caesium based intracellular pipette solutions). The osmolarity of all intracellular pipette solutions was 280-290mOsm and in the case of solution 3, the desired osmolarity was obtained by the addition of sucrose.

**Table 1:** Intracellular solutions.

Number	1	2	3	4	5	6	7	8
Name	K-Gluconate	K-Gluconate + DNDS	K-Gluconate + DNDS + CaCl_2_	K-Gluconate + picrotoxin	Cs-Fluoride + DIDS	K-Gluconate + DIDS	K-Gluconate no ATP/GTP	K-Fluoride
**[K-Gluconate]**	135	135	70	135		135	135	135
**[Cs-Fluoride]**					120			
**[K-Fluoride]**								
**[NaCl]**	8	8		8		8	8	8
**[KCl]**			45		10			
**[HEPES]**	10	10	10	10	10	10	10	10
**[EGTA]**	0.2	0.2		0.2	5	0.2	0.2	0.2
**[MgATP]**	2	2	4	2		2		
**[NaGTP]**	0.3	0.3	0.4	0.3		0.3		
**[CaCl_2_]**			5					
**[Phosphocreatine]**			5					
**[DNDS]**		0.5	0.5					
**[DIDS]**					1	1		
**[Picrotoxin]**				5				

Concentrations in mM

Recordings were made using a Multiclamp 700A amplifier (Molecular Devices). Data was filtered between 0.1Hz-1kHz (or <4kHz) and digitised at 5kHz (or 10kHz) for extracellular (or intracellular patch) recordings, using a power 1401 board (Cambridge Electronic Design, CED). Data was acquired in Spike2 version 7.10 (CED) for sharp wave experiments, or Signal 5 (CED) for all other recordings. Sharp waves were recorded extracellularly from the CA3 pyramidal cell layer. For intracellular patch recordings, CA1 pyramidal cells were held in voltage clamp and series resistance was monitored throughout; cells with a series resistance >40MΩ or with a >30% change during the course of the experiment were discarded from subsequent analysis. Membrane voltage was not corrected for the liquid junction potential. Monosynaptic excitatory and inhibitory synaptic currents (EPSCs and IPSCs respectively) were evoked using 100μs square wave voltage steps through a bipolar tungsten stimulating microelectrode (MicroProbes), placed in the stratum radiatum of CA1, close to the recorded cell. Stimulation intensities had a median value of 5.45V, with an interquartile range of 4-7V. Recordings of evoked IPSCs commenced 1 minute after membrane rupture and whole cell patch clamp recording. IPSCs were recorded in the presence of NBQX disodium salt (NBQX, 10μM) and D-AP5 (50μM) to block AMPA and NMDA receptors, respectively. IPSC amplitudes on average had a median value of 109pA, with an interquartile range of 78.2–142.5pA. The GABA_A_ receptor antagonist, picrotoxin (50μM) was applied to the bath at the end of all experiments measuring inhibitory synaptic transmission to confirm IPSCs were mediated by GABA_A_ receptors. Evoked EPSCs were recorded in the presence of 50μM picrotoxin. NBQX (10μM) and D-AP5 (50μM) were added to the bath at the end of the experiments to confirm the evoked responses were glutamatergic.

### Chemical Analysis

Nuclear magnetic resonance spectra were obtained using deuterium oxide (D_2_O) as the solvent. Proton nuclear magnetic resonance (^1^H NMR) was recorded on a Varian 400-MR spectrometer (400 MHz). Residual non-deuterated solvent was used as an internal standard. Chemical shifts (δ) are quoted in parts per million (ppm). Coupling constants (J) are quoted in Hertz (Hz) and signal splitting patterns are expressed as singlets (s), doublets (d) or doublets of doublets (dd). Carbon nuclear magnetic resonance (^13^C NMR) was recorded on the above-mentioned spectrometer at 100 MHz. The mass spectra were recorded on a MicrOTOF II spectrometer. The mode of ionisation was positive electrospray. Microanalysis was carried out on a CHN Elemental Analyser and a Coulomat 702 C/S Analyser.

### Data Analysis

#### IPSC analysis

The amplitudes of consecutive IPSCs were averaged every minute, and then normalised to the baseline amplitude. Traces, used for examples, were averaged over 1 minute periods.

#### Sharp wave analysis

For sharp wave detection, the extracellular local field potential (LFP) was first DC removed in Spike2 with a time constant of 0.1s. Analysis was then conducted using custom written scripts in Matlab 2012a (Mathworks). Data were downsampled to 1kHz and low pass filtered at <30Hz. Sharp waves were detected when the filtered data exceeded 1.5 SD above the mean for time (t) 10ms≤t≤100ms. Sharp wave boundaries were defined when the data dropped below 0.5 SD above the mean and events were discarded if their amplitude (a) was 20μV≤a≤1mV.

For detection of postsynaptic currents (PSCs) in sharp waves, voltage-clamp data were downsampled to 1kHz, bandpass filtered between 0.5-400Hz and rectified. Analysis times were centred within 100ms windows of each sharp wave peak. PSCs in sharp waves were then detected when the filtered and rectified data exceeded 2 SD above the mean for time 8ms≤t≤300ms. If PSCs were <10ms apart and the length boundaries still held true, PSCs were combined. PSC boundaries were defined when the rectified time derivative of the current signal dropped below 10pA/ms.

#### Statistical analysis

Data are described and plotted as the mean ± SEM. Statistical tests were performed in GraphPad Prism 5 and were chosen following an initial test for normality, with a Kolmogorov-Smirnov test. Details of individual statistical tests are presented in the results section. The level of significance was set at P < 0.05. Calculated probabilities are presented in figures with asterisks denoting the following * P < 0.05, ** P < 0.01, and *** P < 0.001.

### Drugs

NBQX and D-AP5 were purchased from Abcam; Picrotoxin and DIDS were purchased from Sigma-Aldrich; and DNDS was purchased from Fluorochem Ltd or Sigma.

## Results

### Comparison of the effects of intracellular CsF-DIDS, DNDS and picrotoxin on evoked IPSCs

To characterise the efficacy of intracellular agents in blocking evoked and spontaneous IPSCs, we first evoked IPSCs in whole cell patch clamp recordings, from CA1 pyramidal cells, in acutely prepared hippocampal slices. IPSCs were pharmacologically isolated by bath application of 50μM AP5 and 10μM NBQX to block NMDA, AMPA and Kainate receptors and IPSCs were recorded at a holding potential of -50mV, whilst stimulating in stratum radiatum. After a period of at least 20 minutes following membrane rupture to allow diffusion of agents from the pipette solution into the neuron, 50μM picrotoxin was bath applied to confirm the IPSCs were mediated by GABA_A_ receptors. We initially demonstrated that evoked IPSCs were stable over time, when recorded during the diffusion of standard K-Gluconate intracellular pipette solution (intracellular pipette solution 1, Table 1). There was no difference between the normalised evoked IPSC amplitude in the last minute of wash-in compared to the first minute (Fig 1A and 1F; 0.966 ± 0.108, one-sample t-test, P = 0.762, n = 6).

Next we examined the ability of intracellular DNDS (intracellular pipette solution 2, Table 1) to block evoked IPSCs. In contrast to work initially characterising this agent in layer IV visual cortex neurons [48] and a few subsequent studies using this agent to block IPSCs in the barrel [52] and auditory [57] cortices, amygdala [49, 58] and hippocampus [53], we found 500μM DNDS to be ineffective at blocking evoked IPSCs in CA1 pyramidal cells, over a 20 minute time period (Fig 1B and 1F, normalised amplitude in last minute of wash-in: 1.01 ± 0.0584, one-sample t-test, P = 0.818, n = 5). Experiments were conducted at 32°C, increasing the rate of diffusion compared to room temperature, and previous studies showed an effect within 5–10 minutes [53]. The lack of effect of DNDS was consistent across 5 recorded cells, from separate slices, which had a range of initial series resistances from 19.4 to 31.5 MΩ. To rule out the possibility that the absence of an effect was due to the structure and purity of DNDS, we conducted a series of chemical analyses on the compound. Nuclear magnetic resonance and mass spectrometry analyses returned results consistent with the structure of DNDS, matching a previously published spectrum (SDBSWeb: http://sdbs.db.aist.go.jp (National Institute of Advanced Industrial Science and Technology, Japan)), [(^1^H NMR (400 MHz, D_2_O) δ 8.05 (d, *J* = 8.8, 2H), 8.07 (s, 2H), 8.36 (dd, *J* = 8.8, 2.4, 2H), 8.65 (d, *J* = 2.4, 2H); ^13^C NMR (100 MHz, D_2_O) δ 122.5, 126.2, 128.9, 130.1, 140.5, 141.5, 146.3; MS (ES+) m/z 497 (M + Na, 100%)] (see S1 Fig) and the purity of the compound was determined by CHN analysis (calculated for C_14_H_8_N_2_Na_2_O_10_S_2_. 3.3 H_2_O, C, 31.50, H, 2.76, N, 5.25, S, 12.01; found C, 31.56, H, 2.72, N, 5.23, S, 11.96). DNDS being a yellow solid, the presence of the compound in the intracellular solution was indicated by a colour change from colourless to yellow, and a clear solution was observed at all times, showing the solubility of the compound.

Previous work has used DNDS in conjunction with a high intracellular chloride and calcium concentration, and addition of phosphocreatine, to block IPSCs in hippocampal CA1 neurons [53]. To probe whether any of these additional modifications may have altered the apparent efficacy of DNDS we adjusted the intracellular pipette solution to include these modifications whilst maintaining a holding potential of -70mV (intracellular pipette solution 3, Table 1, [53]). This intracellular pipette solution also used DNDS from an alternative supplier (Sigma) which nuclear magnetic resonance analysis showed to be identical to the Fluorochem compound. A small depression in evoked IPSC amplitude was observed 20 minutes after intracellular perfusion with this intracellular pipette solution (Fig 1C and 1F, normalised amplitude in last minute of wash-in: 0.867±0.0505, one-sample t-test, P = 0.0302, n = 9), but the extent of the depression in evoked IPSCs was much less than the block by extracellularly applied picrotoxin (normalised amplitude in last minute of wash-in vs. in last minute of picrotoxin: 0.883±0.171 vs 0.102±0.0179, paired t-test, P<0.0001, n = 7).

We then tested picrotoxin, which was initially characterised to block chloride channels from the intracellular side in bullfrog dorsal root ganglion cells [40, 47] but has been used most widely to block GABA_A_ receptors extracellularly. It has also been used intracellularly in a few studies to block GABA_A_ receptors in the auditory cortex [41, 43, 46], visual cortex [44, 45, 54] and somatosensory cortex [42]. A wide range of picrotoxin concentrations have previously been used with typical concentrations of 1-5mM [41, 43–45, 54]. Therefore we chose to use the upper limit of this concentration range (5mM) to maximise any observable effect. Notably this is 100-fold higher than the concentration of bath-applied picrotoxin, used in our experiments, and so the application of positive pressure as a cell was approached, during patching, was sufficient to block evoked IPSCs once whole-cell recording was obtained. This precluded measurement of the effect of intracellular picrotoxin, therefore, following some precedent [42], we tip-filled our recording pipettes with standard K-Gluconate intracellular pipette solution. Utilising this approach, we still observed an increase in the evoked IPSC amplitude during the initial diffusion of K-Gluconate + picrotoxin (intracellular pipette solution 4, Table 1), as the local extracellular concentration of picrotoxin returned to zero (Fig 1D and 1F). This indicated that the intracellular pipette solution containing picrotoxin reached the pipette tip during the approach to the cell. Following washout of extracellular picrotoxin, the evoked IPSC amplitude plateaued at an elevated level (normalised amplitude in last minute of wash-in: 1.88 ± 0.296, one-sample t-test, P = 0.0208, n = 8) and was subsequently blocked by bath application of 50μM picrotoxin. The stable plateau IPSC amplitude 20 minutes after membrane rupture indicates that intracellular picrotoxin is ineffective at blocking evoked IPSCs.

A caesium fluoride based intracellular pipette solution, containing DIDS (intracellular pipette solution 5, Table 1), has also been used previously to block IPSCs in the visual cortex [54] and hippocampus [53, 56]. Using this intracellular pipette solution, we indeed observed a substantial, although incomplete, blockade of evoked IPSCs during the diffusion of Cs-Fluoride +DIDS when cells were maintained at a holding potential of -80mV (Fig 1E and 1F; normalised amplitude in last minute of wash-in: 0.371 ± 0.0634, one-sample t-test, P<0.0001, n = 7). This indicated that Cs-Fluoride + DIDS might be the most effective intracellular pipette solution to block IPSCs and we next investigated which component of the solution caused the blockade.

### Mechanism of Cs-Fluoride DIDS blockade of evoked IPSCs

The partial blockade of evoked IPSCs by Cs-Fluoride DIDS intracellular pipette solution could emerge from several potential sources: the presence of caesium, which blocks GABA_B_ receptor-mediated IPSCs [59]; DIDS, which blocks chloride channels and GABA responses in certain preparations [55, 60–62]; the absence of nucleotides which can cause a run-down of GABA_A_ responses in acutely dissociated neurones [63–65]; and the presence of fluoride ions, the halogen ion to which GABA_A_ receptors are the least permeable [66, 67] and which has therefore been used in combination with an absence of intracellular nucleotides to block GABA_A_ receptors [67–70].

**Fig 2:**
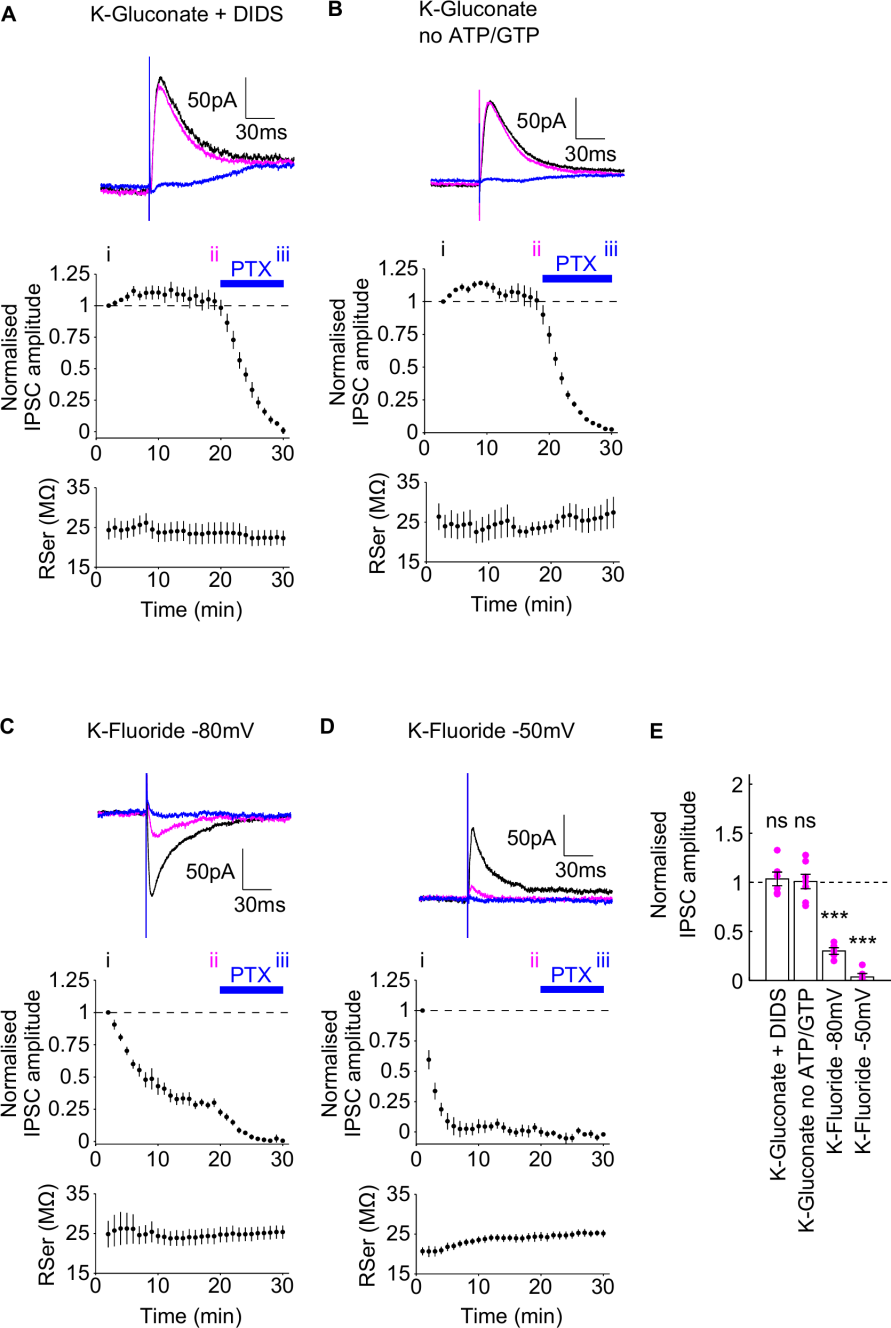
Effect of DIDS, the absence of nucleotides and KF on evoked IPSCs. Upper panels of A-D: Example traces during the diffusion of three intracellular pipette solutions (K-Gluconate +DIDS (A), K-Gluconate without ATP or GTP (B), and K-Fluoride at -80mV (C) and -50mV (D)). Each trace shows the IPSC at the start of recording (black, time point i in middle panels), in the last minute before bath application of picrotoxin (magenta, time point ii in middle panels) and at the end of the bath application of picrotoxin (blue, time point iii in middle panels). Middle panels of A-D: Normalised IPSC amplitudes. Blue bar indicates the presence of bath picrotoxin. Lower panels of A-D: Corresponding series resistance during experiments. NBQX and AP5 were present throughout the experiments. E) Group data of the normalised IPSC amplitudes, taken at time point ii, individual data points marked with magenta dots. Summary statistics represent tests comparing the normalised evoked IPSC amplitudes at time point ii with the normalised baseline amplitude for each data set. Data are plotted as mean ± SEM.

Therefore to further explore which factor in the Cs-Fluoride + DIDS intracellular pipette solution was the most pertinent in contributing to the partial blockade of evoked IPSCs, we first examined whether 1mM DIDS alone could block evoked IPSCs. Using K-Gluconate + DIDS (intracellular pipette solution 6, Table 1), we found the presence of DIDS did not reduce the amplitude of evoked IPSCs (Fig 2A and 2E; normalised amplitude in last minute of wash-in: 1.035 ± 0.0696, one-sample t-test, P = 0.6382, n = 6). The absence of intracellular ATP and GTP (intracellular pipette solution 7, Table 1) also had no effect on evoked IPSC amplitudes (Fig 2B and 2E; normalised amplitude in last minute of wash-in: 1.009 ± 0.0736, one-sample t-test, P = 0.907, n = 7), suggesting the previously observed run-down [63–65] may be sensitive to the type of neuronal preparation. In contrast, the presence of fluoride ions (intracellular pipette solution 8, Table 1) caused a decrease in the evoked inward IPSC amplitude when cells were maintained at a holding potential of -80mV (Fig 2C and 2E; normalised amplitude in last minute of wash-in: 0.301 ± 0.0337, one-sample t-test, P<0.0001, n = 5) which was not significantly different to the depression in the Cs-Fluoride + DIDS intracellular pipette solution (0.301 ± 0.0337 vs 0.371 ± 0.0634, t-test, P = 0.405). Fluoride ions also virtually abolished the evoked outward IPSC amplitude when cells were held at -50mV (Fig 2D and 2E; normalised amplitude in last minute of wash-in: 0.0359 ± 0.0359, one-sample t-test, P<0.0001, n = 5). Intracellular pipette solution 8 also contained no nucleotides because the addition of KF to a MgATP-containing solution can result in the formation of insoluble magnesium fluoride. However, given the lack of any effect by the absence of intracellular nucleotides alone, we directly attribute the effect of intracellular Cs-Fluoride + DIDS on evoked IPSC amplitude to the presence of fluoride ions.

### Intracellular fluoride can block spontaneous IPSCs occurring in hippocampal sharp waves

**Fig 3:**
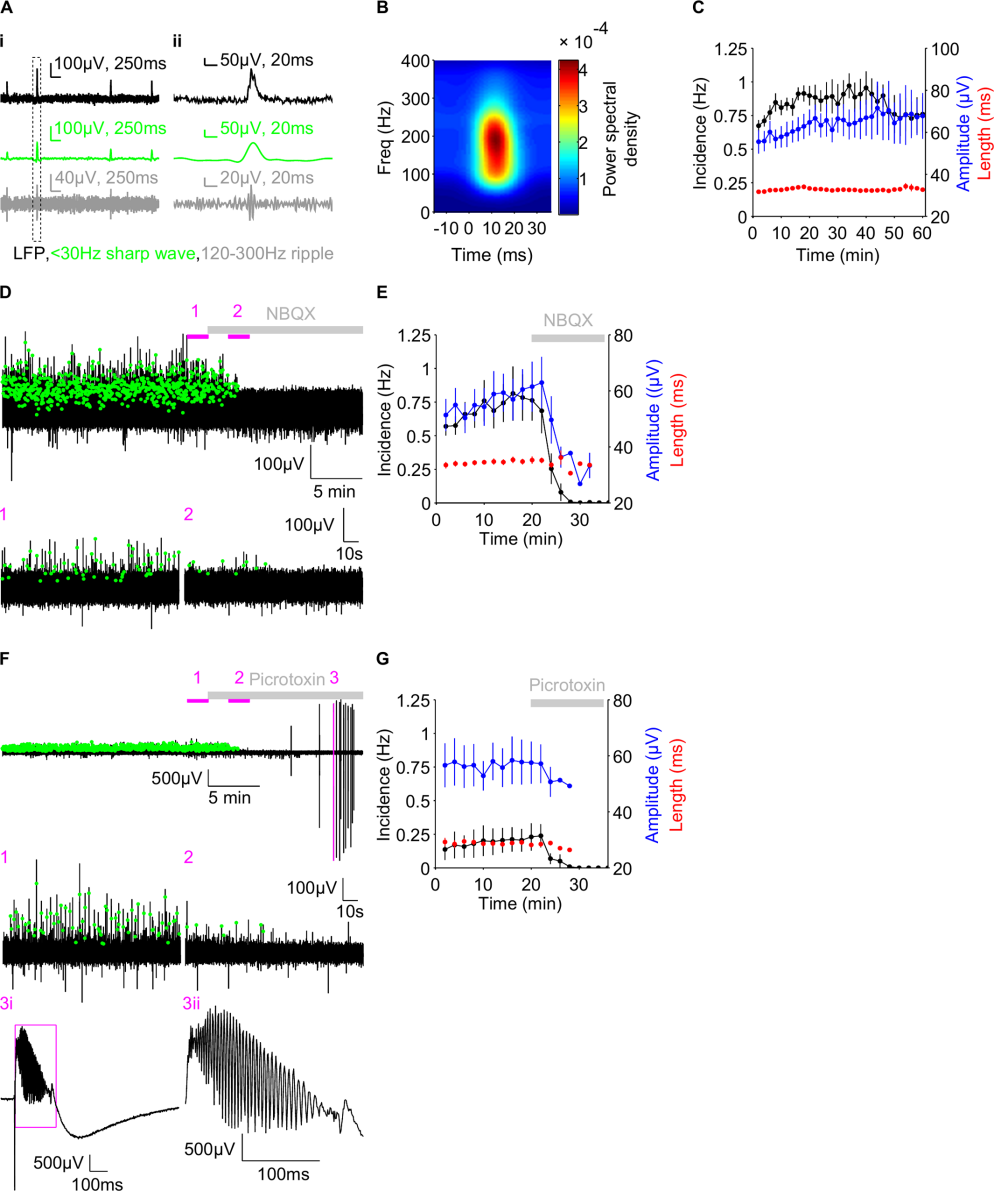
Hippocampal sharp wave characterisation. A) Example trace of hippocampal sharp waves (i) showing the raw local field potential (upper), the filtered <30Hz sharp wave band (middle) and the filtered 120-300Hz ripple band (lower). A zoomed version of a single sharp wave, demarked by the dotted box in i), is shown in ii). B) Average sharp wave spectrogram showing the power (in colour) of different frequency bands with time. C) Group data of the incidence (black), amplitude (blue) and length (red) of sharp waves, in minute bins, over an hour long recording period. D&F) Example trace (upper) before and after NBQX (D) and picrotoxin (F), of the raw LFP, with detected sharp waves demarked by green dots. Magenta bars indicate the regions shown in the zoomed traces (below). F3ii) Further zoom of the magenta box in F3i. Grey bars in D-G indicate the presence of NBQX (D-E) and picrotoxin (F-G). E &G) Group data of sharp wave properties, in minute bins, before and after NBQX (E) and picrotoxin (G). Data are plotted as mean ± SEM.

Having determined that intracellular fluoride was the most effective method to block evoked IPSCs, we next sought to determine whether a fluoride-based intracellular pipette solution could block spontaneous IPSCs, during ongoing patterns of network activity. For this, we chose an *in vitro* model of hippocampal sharp wave ripples [71, 72], whereby sharp waves can be recorded from the CA3 pyramidal cell layer (Fig 3A and 3B), whilst whole cell recordings are obtained from CA1 pyramidal cells. Extracellularly recorded sharp waves were stable over time, for at least an hour (Fig 3C), with no differences between the last 5 minutes of each quarter of the hour for sharp wave incidence (1^st^ quarter 0.839 ± 0.0521Hz, 2^nd^ quarter 0.864 ± 0.131Hz, 3^rd^ quarter 0.892 ± 0.109Hz, 4^th^ quarter 0.751 ±0.129Hz, repeated measures ANOVA, P = 0.607, n = 6 slices), amplitude (1^st^ quarter 56.7 ± 4.59μV, 2^nd^ quarter 60.8 ± 6.17μV, 3^rd^ quarter 64.4 ± 10.1μV, 4^th^ quarter 66.0 ± 9.81μV, Friedman test, P = 0.874, n = 6 slices) nor length (1^st^ quarter 32.0 ± 0.365ms, 2^nd^ quarter 32.0 ± 0.516ms, 3^rd^ quarter 32.2 ± 0.477ms, 4^th^ quarter 32.0 ±0.683ms, repeated measures ANOVA, P = 0.984, n = 6 slices).

Fast glutamatergic and GABAergic transmission are necessary preconditions for the emergence of sharp waves, since sharp waves can be blocked following 10μM NBQX (Fig 3D and 3E, n = 5) or 50μM picrotoxin (Fig 3F and 3G, n = 5) and, in the case of picrotoxin, be replaced by epileptiform activity, as previously described [18, 19, 24, 33, 73–75]. Therefore, in order to isolate excitatory and inhibitory synaptic transmission during sharp wave activity, a suitable intracellular solution is required to block spontaneous IPSCs in the recorded neuron, without perturbing network activity.

**Fig 4:**
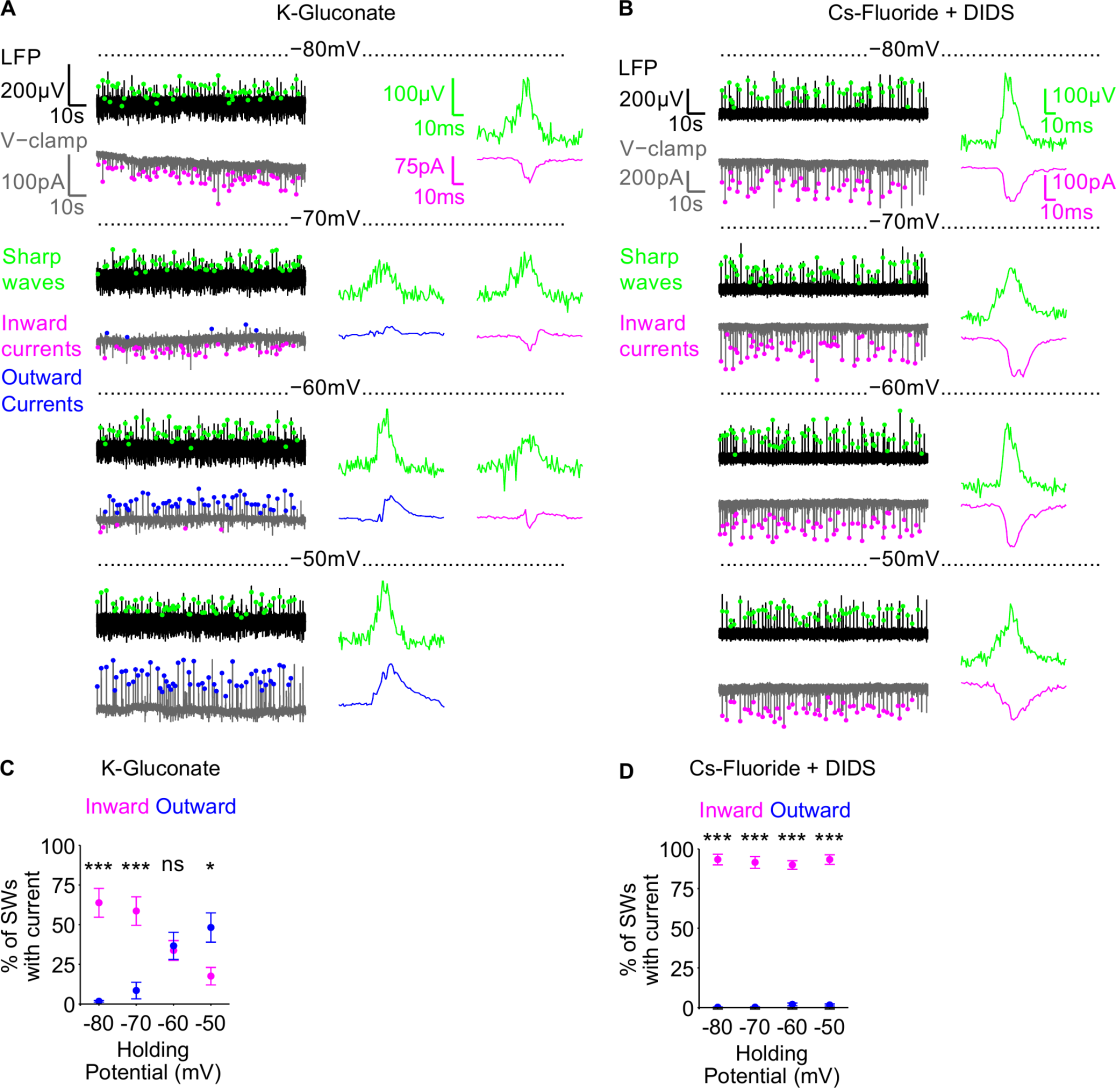
Fluoride-based intracellular pipette solution can block spontaneous IPSCs during hippocampal sharp waves. A-B) Example traces of an experiment using K-Gluconate (A) and Cs-Fluoride +DIDS intracellular pipette solutions (B) are shown in the left panels. In each case, the LFP (black) was recorded whilst holding the cell at 1 of 4 different holding potentials (-80mV, -70mV, -60mV and -50mV) (grey). Inward and outward PSCs, detected during sharp waves (green dots), are demarked by magenta and blue dots respectively. Individual example sharp waves and PSCs, at the different holding potentials, are shown in the right panels. C-D) Group data showing the percentage of sharp waves for which a concomitant inward (magenta) or outward (blue) PSC was detected, at the different holding potentials, in K-Gluconate (C) and Cs-Fluoride + DIDS (D) intracellular pipette solutions. Data are plotted as mean ± SEM.

We assessed whether a fluoride-based intracellular pipette solution could serve this purpose by recording the spontaneous postsynaptic currents (PSCs), impinging onto CA1 pyramidal cells in voltage clamp, during the ongoing sharp waves, recorded in CA3 (Fig 4). Under these conditions, at each holding potential used (-80mV, -70mV, -60mV and -50mV), excitatory currents are inward, whereas inhibitory currents are inward at holding potentials more hyperpolarised than the inhibitory reversal potential (-72.3mV in K-Gluconate intracellular pipette solution) and outward at holding potentials more depolarised than the inhibitory reversal potential. Therefore, in the absence of inhibition blockade, both EPSCs and IPSCs could impinge onto CA1 pyramidal cells, during sharp waves, and the direction of observed PSCs within sharp waves will likely reverse around the inhibitory reversal potential. Conversely, with inhibition blocked, PSCs should remain inward across the range of membrane potentials. Using standard, K-Gluconate intracellular pipette solution, we found there was a significant holding potential by PSC direction interaction on the proportion of sharp waves with a detectable PSC (Fig 4A and 4C; 2-way repeated measures ANOVA, P< 0.0001, n = 15 cells, from 9 slices), with a main effect of PSC direction (P = 0.0201) but not holding potential (P = 0.965). At -80mV and -70mV there were a greater proportion of sharp waves with a detectable inward PSC than outward PSC (-80mV: inward 63.8 ± 9.08% vs outward 1.71 ± 0.538%, P<0.001; -70mV: inward 58.6 ± 8.98% vs outward 8.48 ± 5.23%, P<0.001), at -60mV the proportions were not different (inward 33.7 ± 6.25% vs outward 36.6 ± 8.55%, P>0.05), and at -50mV there was a greater proportion of sharp waves with an outward PSC than inward PSC (inward 17.5 ± 5.51% vs outward 48.2 ± 9.26%, P<0.05). The switch from predominantly inward to predominantly outward currents suggests that, in agreement with previous studies [73, 74, 76], the dominant PSC onto CA1 pyramidal cells during the sharp waves are IPSCs.

If a fluoride-based intracellular pipette solution can block spontaneously occurring IPSCs, then the Cs-Fluoride + DIDS intracellular pipette solution should unmask a similar proportion of detectable inward PSCs in sharp waves across all holding potentials. Consistent with this hypothesis, we observed no interaction between holding potential and PSC direction on the proportion of sharp waves with a detectable current (Fig 4B and 4D; 2-way repeated measures ANOVA, P = 0.3984, n = 13 from 6 slices), with a main effect of PSC direction (P<0.0001) and no effect of holding potential (P = 0.708). Across all holding potentials, there were a greater proportion of inward than outward PSCs (-80mV: inward 93.3 ± 3.37% vs outward 0.197 ± 0.197%, P<0.001; -70mV: inward 91.5 ± 3.70% vs outward 0.240 ± 0.240%, P<0.001; -60mV inward 89.9 ± 2.71% vs outward 2.00 ± 0.865%, P<0.001; -50mV: inward 93.3 ± 3.02% vs outward 1.64 ± 0.653%, P<0.001), consistent with the conclusion that spontaneously occurring ISPCs had been blocked, and EPSCs were revealed.

### Fluoride ions depress spontaneous and evoked EPSCs

**Fig 5:**
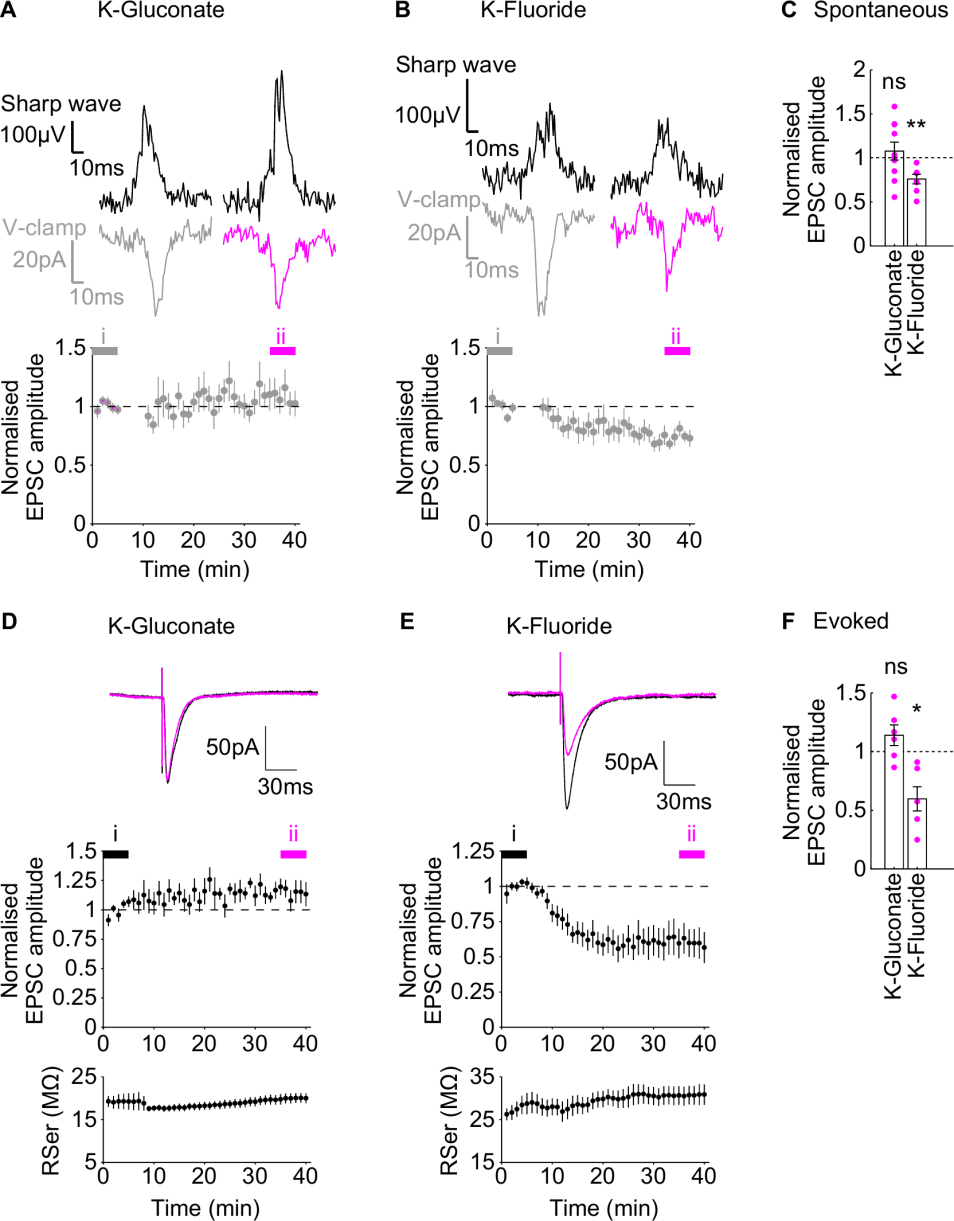
Effect of Fluoride on spontaneous and evoked EPSCs. A-B) Example traces (upper panel) of sharp waves (black) and corresponding spontaneous EPSCs from the 1^st^ (left, grey) and last (right, magenta) 5 minutes of recording in K-Gluconate (A) and K-Fluoride (B) intracellular pipette solutions. Lower panel: Group normalised EPSC amplitude time course, during wash-in. Grey bar (time point i) and magenta bar (time point ii) indicate the times during which the example traces in the upper panels are taken from. Upper panels of D-E): Example traces of pharmacologically isolated, evoked EPSCs during the wash-in of K-Gluconate (D) and K-Fluroride (E). Each trace shows the EPSC at the start of wash-in (black, time point i in middle panels) and at the end of the wash-in period (magenta, time point ii in middle panels). Middle panels of D-E: Normalised EPSC amplitudes during wash-in. Lower panels of D-E: Corresponding series resistance during wash-in. Picrotoxin was present in the bath throughout the experiments. C&F) Average group data of the normalised EPSC amplitudes, taken in the last 5 minutes of recording for the spontaneous (C) and evoked (F) EPSCs, individual data points marked by magenta dots. Summary statistics represent tests comparing the normalised EPSC amplitudes in the last 5 minutes of recording with the normalised baseline amplitude for each data set. Data are plotted as mean ± SEM.

Our data show that the introduction of fluoride ions intracellularly is effective to block inhibitory synaptic inputs in a cell-specific manner. However, it is not clear what effects fluoride ions might have on other cell properties. To address this, we examined whether the introduction of fluoride would affect the amplitude of spontaneously occurring EPSCs, impinging onto CA1 pyramidal cells during ongoing sharp waves. EPSCs were isolated by holding the cell at the inhibitory reversal potential (-70mV). Using the standard K-Gluconate intracellular pipette solution, the normalised, spontaneous EPSC amplitude, 35–40 minutes after going whole cell, was not significantly different from the baseline first 5 minutes (Fig 5A and 5C; normalised amplitude at 35–40 min: 1.08 ± 0.102, one-sample t-test, P = 0.468, n = 10) whereas the K-Fluoride intracellular pipette solution caused a depression in the spontaneous EPSC amplitude (Fig 5B and 5C; normalised amplitude at 35–40 min: 0.741 ± 0.0550, one-sample t-test, P = 0.0033, n = 7). Since fluoride ions block IPSCs occurring during sharp waves this would likely increase the observed EPSC amplitude due to the reduction in shunting. Therefore, it is likely that the observed depression in spontaneous EPSC amplitude is an underestimate of the actual effect of fluoride ions.

We confirmed that the K-Fluoride intracellular pipette solution depresses EPSCs by recording pharmacologically isolated, evoked EPSCs. In the presence of 50μM bath picrotoxin, evoked EPSCs were stable when recorded in K-Gluconate intracellular pipette solution at a holding potential of -70mV (Fig 5D and 5F; normalised EPSC amplitude at 35–40 min: 1.14 ± 0.0879, one-sample t-test, P = 0.173, n = 6). However during diffusion of the K-Fluoride, evoked EPSCs depressed (Fig 5E and 5F; normalised EPSC amplitude at 35–40 min: 0.598 ± 0.103, one-sample t-test, P = 0.0113, n = 6). Therefore a fluoride-based intracellular pipette solution does not selectively block IPSCs since it also substantially inhibits EPSCs.

## Discussion

A method to selectively block inhibitory synaptic transmission in a cell-specific manner would be a valuable tool to dissect the circuit dynamics that occur during ongoing network activity. The principal but rarely used method to achieve this goal is the inclusion of agents in the intracellular media during whole cell patch clamp or sharp electrode recording with glass pipettes. In this study we systematically compared the efficacy of three of the most commonly used agents: DNDS, picrotoxin and Cs-Fluoride + DIDS. We found Cs-Fluoride + DIDS is the most effective method for blocking spontaneous and evoked IPSCs onto CA1 pyramidal cells, exerting a partial blockade, whereas DNDS and picrotoxin are mostly ineffective (Fig 1). Further analysis revealed that fluoride and not DIDS is the important component of Cs-fluoride + DIDS for this blockade but intracellular fluoride ions also cause a depression in EPSC amplitude. Our studies therefore show that fluoride ions are the most effective agent for intracellular blockade of IPSCs but their utility is restricted by a lack of selectivity over other cellular processes.

A number of studies have used different intracellular agents to block inhibitory synaptic transmission with varying efficacies but none have directly compared the available methods. In highlighting their relative efficacies we also reveal some discrepancies between our results and previous studies.

Picrotoxin has been widely used as an extracellular blocker of GABA_A_ receptors where it acts as a non-competitive pore blocker. Picrotoxin has also been shown to block GABA_A_ receptors from the intracellular side of the membrane in bullfrog dorsal root ganglia cells by bath application of picrotoxin to inside-out patches [40, 47]. Since then, a few studies have used intracellular picrotoxin to block IPSCs [41–45]. Our experiments demonstrate that the interpretation of intracellular picrotoxin blockade is not straightforward since the diffusion of picrotoxin from the patch/sharp electrode into the extracellular domain before a single cell recording is obtained can substantially inhibit GABA_A_ receptors. This is especially important, given that the concentration of picrotoxin used (1-5mM) is often considerably greater than the IC_50_ to reduce GABA_A_ receptor-mediated currents [77–80]. By recording evoked IPSCs continuously from the moment of membrane rupture, it is possible to discriminate between an intracellular and an extracellular effect.

Namely, if IPSCs are present initially and then their amplitude decreases, the effect of picrotoxin can be attributed to an intracellular block. In contrast, if IPSCs are blocked or are small initially and their amplitude then increases, the effect of picrotoxin can be attributed to an extracellular block. We observed the latter scenario, indicating the effects of picrotoxin were primarily mediated through extracellular, rather than intracellular, blockade. We note that at lower concentrations of picrotoxin, the same reasoning may not apply, see [25, 42]. The absence of an intracellular effect of picrotoxin cannot be due to different GABA_A_ receptor subunit compositions in the cells recorded here, compared to previous studies because, at least in one previous study, the same species, strain and age of mice were used for recordings from CA1 pyramidal cells [25].

The disulphonic stilbene derivatives DNDS and DIDS are thought to be chloride channel blockers that therefore inhibit GABA_A_ receptors but they also have wider effects on chloride homeostasis [55, 60–62, 81–84]. Intracellular DNDS has been shown to inhibit the response to bath applied muscimol [57] and shift current-voltage relationship for mixed excitatory and inhibitory synaptic responses [52] in comparison to recordings without DNDS. In addition, within cell comparisons of DNDS blockade of IPSCs/IPSPs show a time-dependent blockade commensurate with the diffusion of DNDS to synaptic locations [48, 53]. In one of these studies, the blockade by DNDS was facilitated by the inclusion of intracellular CaCl_2_ and phosphocreatine [53]. Even when we added these agents intracellularly we could see only a limited effect of DNDS on evoked IPSCs. The reason for this discrepancy is not immediately apparent and is unlikely to be due to different GABA_A_ receptor subunit compositions because recordings were made from CA1 pyramidal cells, in the same species, strain and age of mice [53]. However, we note that other studies have also failed to find effectiveness for DNDS [85]. It is possible that non-specific effects of DNDS on chloride homeostasis [83, 84] may play a role whereby DNDS changes the intracellular chloride concentration producing an apparent depression of GABA_A_ receptor-mediated currents under certain experimental conditions. It is also possible that different sources of DNDS may explain the discrepancies. We used DNDS from two separate sources (Sigma and Fluorochem), whose molecular structure was confirmed by nuclear magnetic resonance and mass spectrometry, whereas other studies used DNDS provided by the researchers at the University of Pittsburgh [48, 53]. We also cannot exclude the possibility that higher concentrations of DNDS, to that used here (500μM), might exert stronger effects.

DIDS blocks GABA responses in dorsal root ganglion neurones when applied extracellularly [61] and can block chloride channels [55, 60, 62] although there is no evidence that intracellular DIDS can block GABA_A_ receptor-mediated IPSCs on its own (Fig 2). The robust, albeit incomplete, depression of IPSC amplitudes, using the Cs-Fluoride + DIDS intracellular pipette solution (Fig 1), is consistent with previous work [53, 54, 56] and can be attributed to the presence of fluoride ions, rather than the presence of DIDS or the absence of intracellular nucleotides (Fig 2) [68–70, 85, 86].

We found the timecourse of the blockade to be faster and more complete for outward IPSCs at -50mV (above the chloride reversal potential) compared to inward IPSCs at -80mV (below the chloride reversal potential). The reason behind this difference is unclear and is opposite to that observed previously, where in immature rat CA3 pyramidal neurones inward IPSCs were suppressed prior to outward IPSCs [68]. Previously, the fast blockade of inward IPSCs was attributed to the replacement of intracellular chloride with fluoride [68]. GABA_A_ receptors are least permeable to fluoride ions [66, 67], of the halogen ions, likely due to the large fluoride hydration shell [66]. Hence inward IPSCs, mediated by anion efflux, are minimised following the introduction of fluoride. The larger concentration of intracellular chloride in the intracellular pipette solution here (8mM) compared to previously (2mM, [68]), may explain why the blockade observed here is only partial.

In contrast, outward IPSCs, mediated by the influx of chloride, should be spared following the introduction of fluoride. The longer timecourse for outward IPSC suppression, reported previously [68], was attributed to the run-down of GABA_A_ receptor function in the absence of MgATP [63–65] although our data do not support this (Fig 2B and 2E). It is also possible that fluoride alters the GABA_A_ receptor equilibrium potential. Indeed replacement of intracellular chloride with different anions does cause a shift in the GABA_A_ receptor equilibrium potential [67] which could result in a depression of outward IPSC amplitude if it was shifted to a more depolarised value than -50mV. However, if this were the case, an increase in the amplitude of inward IPSCs would be observed over the same timescale which we did not see. Therefore, the mechanism for fluoride induced IPSC depression is unlikely to be a change in the GABA_A_ receptor equilibrium potential. Moreover, replacement of chloride with fluoride has been shown to shift the GABA_A_ receptor equilibrium potential more hyperpolarised, rather than depolarised [67].

Fluoride ions are likely to have other effects than simply blocking GABA_A_ receptors. For example, sodium fluoride is widely used as a serine-threonine phosphatase inhibitor [87–89] and to activate specific G-proteins [89–91]. We found the K-Fluoride intracellular pipette solution to depress both spontaneous and evoked EPSCs (Fig 5). During these experiments, cells were held at -70mV and therefore the depression of EPSCs is likely mediated by an effect on AMPA receptors, given that NMDARs are not substantially active at this holding potential [92]. This is in contrast to studies on immature rat CA3 pyramidal cells where AMPA receptor-mediated responses were unaffected by a fluoride [68, 69, 86]. This action of fluoride, along with the restriction it makes on using nucleotides in the intracellular solution, places clear restraints on the scope to which a fluoride-based intracellular pipette solution could be used to block inhibition.

The methodology employed to block inhibition is research question specific. In many cases it is sufficient to explore the isolated effects of excitation, or infer the influence of global inhibition, via the use of widespread GABAergic antagonism. Alternatively, genetic-targeting and optogenetic methods, allow dissection of the roles of specific types of interneurones in ongoing behaviours or patterns of activity [24, 32, 33, 93–95]. However, tools to block inhibition in a cell-specific manner can be used to explore the contribution of synaptic connections during patterns of network activity, such as sharp wave ripples, that depend on intact inhibition. This can either be achieved by clamping cells at the inhibitory reversal [53, 76] or by using intracellular agents to block GABA_A_ receptors intracellularly [53]. The latter method avoids the space clamp [35] and shunting inhibition effects [38] that remain using the former method. Our data, and the fact that few groups have used these techniques to date, suggest that further development of these tools is necessary before they may be satisfactorily employed both *in vitro* and *in vivo*.

## Supporting Information

S1 Fig NMR spectra of DNDS (purchased from Fluorochem Ltd.). A) and B) show spectra consistent with the structure of DNDS. A) ^1^H NMR taken on a Varian 400 MR spectrometer in D_2_0 with insert showing close-up of relevant peaks. B) ^13^C NMR taken on a Varian 400 MR spectrometer in D_2_0 with insert of compound structure.
(PDF)
