# Supplementary figures and images for: Assessment of Methods for the Intracellular Blockade of GABAA Receptors
Source: PLoS One. 2016 Aug 8;11(8):e0160900. doi: 10.1371/journal.pone.0160900 (PMC4976935; doi:10.1371/journal.pone.0160900)

A

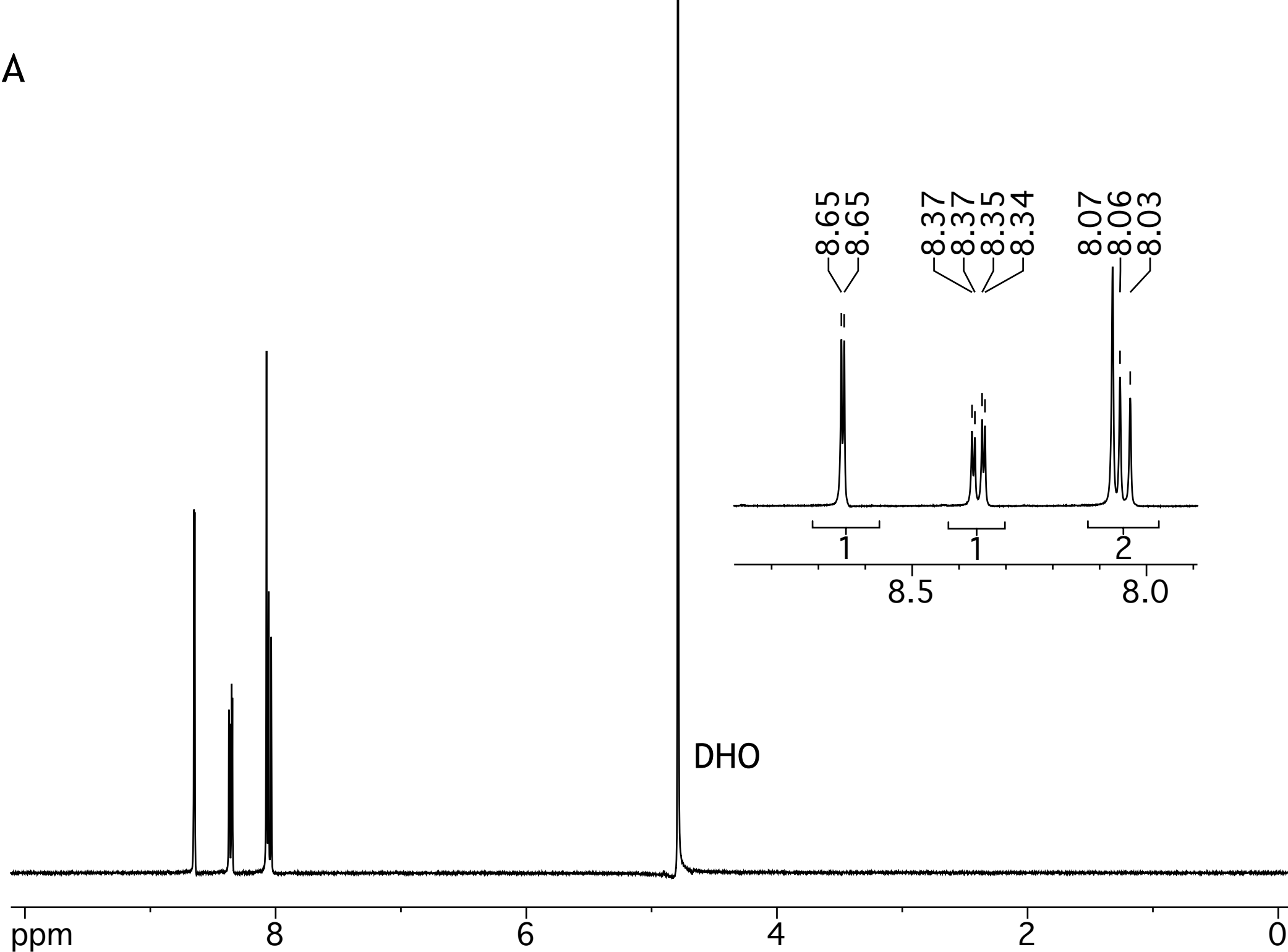

B

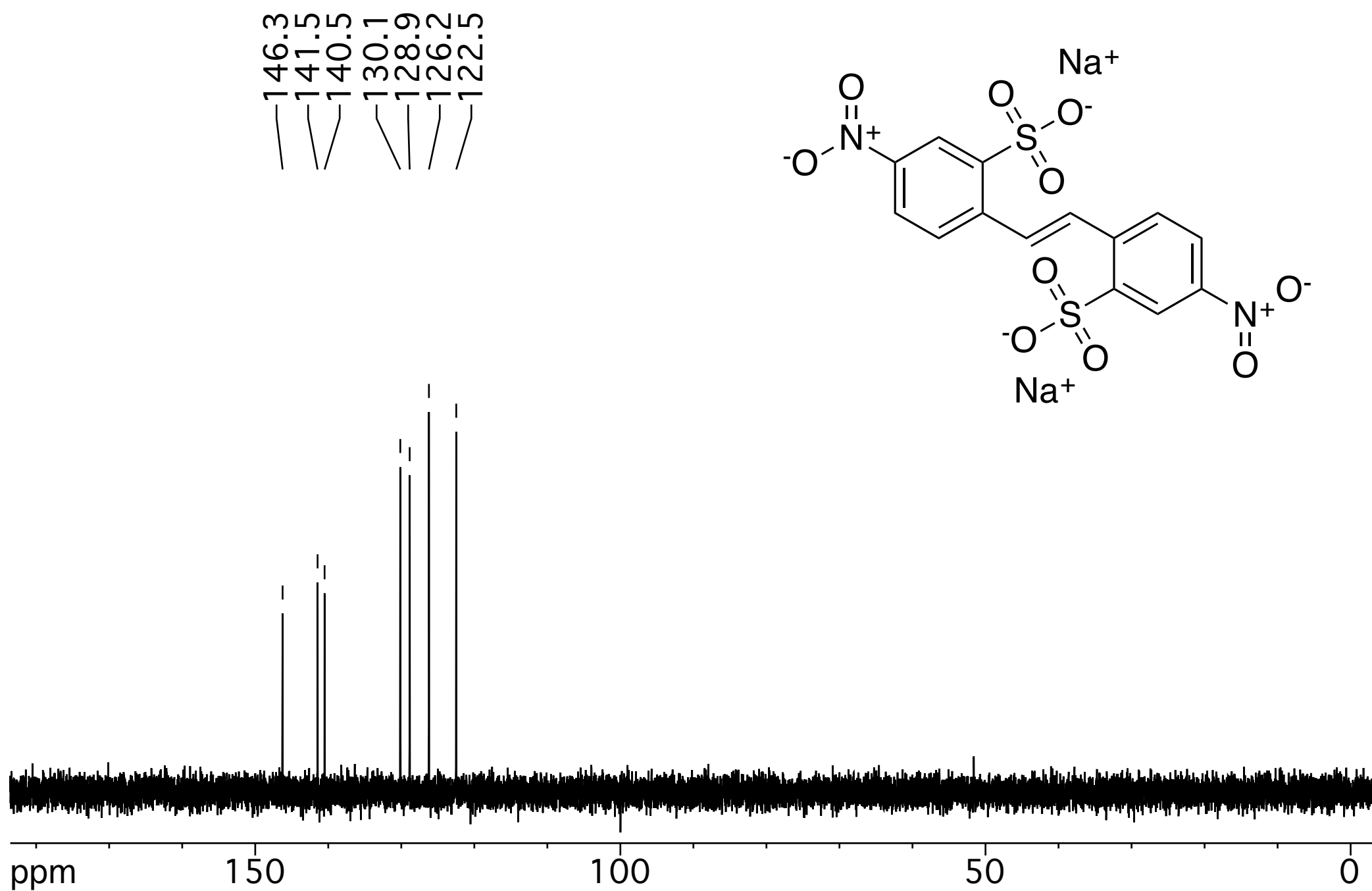

Supplement: S1 Fig — A) and B) show spectra consistent with the structure of DNDS. A) 1H NMR taken on a Varian 400 MR spectrometer in D20 with insert showing close-up of relevant peaks. B) 13C NMR taken on a Varian 400 MR spectrometer in D20 with insert of compound structure. (PDF) [file pone.0160900.s001.pdf]
